# Reduced Risk of Benign Paroxysmal Positional Vertigo in Patients with Parkinson’s Disease: A Nationwide Korean Cohort Study

**DOI:** 10.3390/healthcare13101145

**Published:** 2025-05-14

**Authors:** Dae Myoung Yoo, Ho Suk Kang, Ji Hee Kim, Joo-Hee Kim, Hyo Geun Choi, Kyeong Min Han, Nan Young Kim, Woo Jin Bang, Mi Jung Kwon

**Affiliations:** 1 Hallym Data Science Laboratory, Hallym University College of Medicine, Anyang 14068, Republic of Korea; ydm1285@naver.com (D.M.Y.); km_han@hallym.ac.kr (K.M.H.); 2 Laboratory of Brain and Cognitive Sciences for Convergence Medicine, Hallym University College of Medicine, Anyang 14068, Republic of Korea; 3 Division of Gastroenterology, Department of Internal Medicine, Hallym University Sacred Heart Hospital, Hallym University College of Medicine, Anyang 14068, Republic of Korea; hskang76@hallym.or.kr; 4 Department of Neurosurgery, Hallym University Sacred Heart Hospital, Hallym University College of Medicine, Anyang 14068, Republic of Korea; kimjihee.ns@gmail.com; 5 Division of Pulmonary, Allergy, and Critical Care Medicine, Department of Medicine, Hallym University Sacred Heart Hospital, Hallym University College of Medicine, Anyang 14068, Republic of Korea; luxjhee@gmail.com; 6 Suseo Seoul E.N.T. Clinic, 10, Bamgogae-ro 1-gil, Gangnam-gu, Seoul 06349, Republic of Korea; mdanalytics@naver.com; 7 Hallym Institute of Translational Genomics and Bioinformatics, Hallym University Medical Center, Anyang 14068, Republic of Korea; honeyny78@gmail.com; 8 Department of Urology, Hallym University Sacred Heart Hospital, Hallym University College of Medicine, Anyang 14068, Republic of Korea; 9 Department of Pathology, Hallym University Sacred Heart Hospital, Hallym University College of Medicine, Anyang 14068, Republic of Korea

**Keywords:** Parkinson’s disease, benign paroxysmal positional vertigo, longitudinal follow-up study, national health screening cohort data

## Abstract

**Background/Objectives**: Parkinson’s disease (PD) and benign paroxysmal positional vertigo (BPPV) are both prevalent in the geriatric population. While dizziness is a common non-motor symptom in PD, the relationship between PD and incident BPPV remains unclear. Limited data suggest potential shared mechanisms, including mitochondrial dysfunction and oxidative stress, but large-scale epidemiological evidence is lacking. This investigation focused on assessing the incidence of BPPV in patients with PD compared to matched controls using a nationwide cohort. **Methods**: Data from the Korean National Health Insurance Service–Health Screening Cohort were used to perform a retrospective cohort analysis. We identified 8232 newly diagnosed PD patients and matched them 1:4 with 32,928 controls based on age, sex, income, and residential region. Stratified Cox proportional hazards models were used to estimate hazard ratios (HRs) and 95% confidence intervals (CIs) for incident BPPV. Subgroup and Kaplan–Meier analyses were also performed. **Results**: Over 220,151 person-years of follow-up revealed a lower incidence of BPPV in the PD group relative to the control group (4.98 vs. 5.95 per 1000 person-years); the corresponding adjusted HR was 0.77 (95% CI: 0.66–0.90; *p* = 0.001), indicating a 23% reduced risk. The inverse association remained consistent across most subgroups, including older adults and rural residents. Kaplan–Meier analysis further illustrated a significant decline in the cumulative incidence of BPPV in PD patients (*p* = 0.007). **Conclusions:** PD may contribute to a lower incidence of BPPV, which could be explained by reduced mobility, altered vestibular function, or diagnostic challenges. Clinicians should consider BPPV in PD patients presenting with dizziness.

## 1. Introduction

Parkinson’s disease (PD) is the world’s second leading neurodegenerative disease [1,2,3,4], as its occurrence continues to rise in aging populations, including South Korea [5,6]. The hallmark of PD is the progressive loss of dopaminergic neurons and accumulation of α-synuclein in the brain, which leads to the cardinal motor symptoms of the disease: bradykinesia (a required feature for diagnosis), rigidity, resting tremor, and postural or balance instability that typically appears in the later stages [7]. Beyond these motor features, PD is now recognized as a complex multisystem disorder characterized by diverse non-motor symptoms [8]. These include cognitive impairment, depression, anxiety, sleep disturbances, autonomic dysfunction (e.g., orthostatic hypotension and constipation), and sensory abnormalities [8,9,10]. Among these, dizziness and imbalance—often attributed to autonomic dysfunction such as orthostatic hypotension [9,10]—are increasingly being linked to vestibular dysfunction [11,12,13,14]. These symptoms, though common and disabling, remain under-recognized and may obscure the diagnosis of treatable vestibular conditions such as benign paroxysmal positional vertigo (BPPV) [8,9,10]. Despite emerging evidence implicating vestibular dysfunction in PD, its clinical relevance remains underexplored [11,14].

BPPV, the most frequent vestibular dysfunction of peripheral origin [15,16], occurs most frequently in aging populations, especially women [17]. It may result from displacement of otoconia into the semicircular canals [18], causing vertigo triggered by head movements [19]. Although BPPV is highly treatable using repositioning maneuvers [15,20], it is often underdiagnosed in individuals with limited mobility or cognitive impairment—characteristics frequently present in PD patients [12,21].

Some pathophysiological links between PD and BPPV have been proposed, including mitochondrial dysfunction, oxidative stress, and suppression of SIRT1, a key cellular regulator of aging and stress responses [22,23,24,25]. These shared mechanisms suggest a potential association, but direct epidemiological evidence remains scarce. A small Dutch observational study reported a 5.3% prevalence of BPPV in PD patients, many of whom were previously undiagnosed [26]. However, no large-scale population-based studies have directly compared BPPV incidence between PD patients and matched non-PD controls.

To address this gap, this study utilized a nationwide longitudinal cohort analysis based on the Korean National Health Insurance Service–Health Screening Cohort (KNHIS-HEALS). Our objective was to investigate whether patients with PD have a different risk of developing BPPV compared to matched controls, and to assess whether this association is observed similarly among diverse demographic and clinical populations.

## 2. Materials and Methods

### 2.1. Research Design and Data Collection Source

The present analysis was based on a retrospective cohort design utilizing data from KNHIS-HEALS, a representative national cohort of Korean adults aged 40–79 who took part in health screening assessments from 2002 to 2003 and were monitored until 2019 [27,28]. The database contains demographic, clinical, and lifestyle data for approximately 10% of the Korean population [27,28]. Ethical clearance was granted by the Hallym University IRB (IRB No: 2022-12-005; approval date: 22 December 2022).

**Figure 1:**
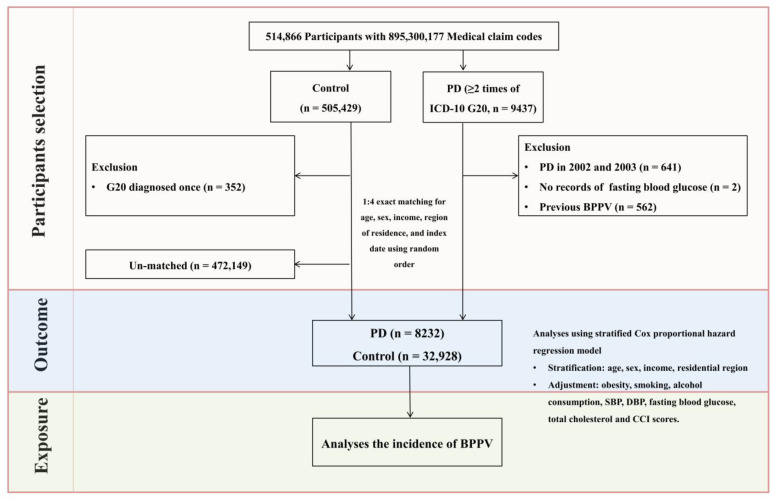
Flowchart outlining the participant selection process. Of the 514,866 eligible individuals, 8232 patients with PD were finally matched with 32,928 control subjects according to age, sex, income, and residential area. The process involved several steps including initial screening, application of inclusion and exclusion criteria, and final matching of patients with controls. And then, BPPV cases were recorded for both groups via ICD-10 codes from the index date to the end of 2019.

To minimize baseline demographic and clinical differences and reduce potential confounding, a 1:4 propensity score matching was performed based on sex, age, income level, and residential area. The chosen ratio reflects existing evidence that higher matching ratios offer diminishing statistical returns [29]. Control participants without PD were randomly selected, and the index date for each individual with PD was defined as the date of their initial diagnosis. For each matched control, the corresponding index date was aligned with that of the matched PD participant. To maintain cohort balance, individuals in the control group who had either died or received a BPPV diagnosis prior to the index date were excluded, leading to the removal of 472,149 control participants during the matching process. All PD cases were successfully matched during the process. While this exclusion reduced the overall number of control subjects, it facilitated the construction of a well-balanced and analytically comparable cohort [29]. The final study population comprised 41,160 participants including 8232 PD group and 32,928 control group. And then, diagnoses of BPPV were monitored in both groups using ICD-10 classification codes, starting from the index date of each participant and continuing through the end of 2019 (Figure 1).

### 2.2. Exposure (PD) and Outcome (BPPV) Definitions

PD was identified based on ICD-10 diagnostic code G20, and only newly diagnosed patients were included—defined as individuals with at least two neurologist-confirmed diagnoses recorded for the first time during the study period—to ensure diagnostic accuracy and temporal alignment with outcome monitoring [30]. BPPV was identified by the presence of at least two treatment claims coded as ICD-10 H81.1, issued by a neurologist or otolaryngologist [31].

### 2.3. Covariates

Sociodemographic variables included age (categorized in 10 five-year groups from 40–44 to ≥85 years), sex, income level (divided into five classes), and residential region (urban vs. rural). Urban areas included major metropolitan cities (e.g., Seoul, Busan), while all other provinces were classified as rural [32].

Lifestyle factors such as smoking status and alcohol consumption were self-reported via health screening questionnaires. Clinical measurements including systolic and diastolic blood pressure, fasting blood glucose, and total cholesterol were obtained from health examination data [27].

Comorbidity burden was assessed using the Charlson comorbidity index (CCI), which encompasses 17 comorbid conditions [33]. Scores ranged from 0 to 29, with higher values indicating greater disease burden [33]. In this study, diabetes (E10–E14) and cerebral stroke (I60–I66) were excluded from the CCI calculation to avoid overlap with potential study outcomes.

### 2.4. Statistical Analyses

Baseline characteristics between PD and control groups were compared using standardized differences, with values <0.2 indicating a negligible imbalance [34]. The association between PD and incident BPPV was assessed using stratified Cox proportional hazards models. Stratification variables included age, sex, income, and residential region. Both crude and adjusted hazard ratios (HRs and aHRs) with 95% confidence intervals (CIs) were estimated. To control for potential confounders, the model included adjustments for weight status, smoking behavior, alcohol consumption, cardiovascular metrics (blood pressure), metabolic indicators (fasting glucose and cholesterol), and overall comorbidity burden as measured by the CCI. Incidence rates per 1000 person-years and incidence rate differences with 95% CIs were also calculated. Subgroup analyses were conducted by age (<75 vs. ≥75 years), sex, income level (low vs. high), and residential region (urban vs. rural). To assess the cumulative incidence of BPPV, survival probabilities were estimated using the Kaplan–Meier method and differences between groups were evaluated using the log-rank test.

Two-sided statistical testing was applied, with significance set at *p* < 0.05. All computations were executed using SAS 9.4 (SAS Institute, Cary, NC, USA).

## 3. Results

### 3.1. Summary of Baseline Features

A total of 41,160 participants were included in the analysis, comprising 8232 individuals with PD and 32,928 matched controls. Due to the matching procedure based on age, sex, income level, and residential region, these characteristics were identically distributed between the two groups, with a standardized difference of 0.00 for each variable (Table 1).

Beyond the matched variables, most baseline health-related characteristics were also well balanced, with absolute standardized differences of less than 0.2, indicating no substantial imbalance between groups. The mean follow-up period was 69.75 months.

### 3.2. Association Between PD and Likelihood of BPPV

Over a total follow-up duration of 220,151 person-years, the incidence rate of BPPV was lower in the PD group compared to the control group (4.98 vs. 5.95 per 1000 person-years), with an incidence rate difference of −0.97 per 1000 person-years (95% CI: −1.81 to −0.14) (Table 2).

In the unadjusted analysis, individuals with PD had a significantly reduced hazard of developing BPPV compared to controls (HR: 0.81, 95% CI: 0.69–0.94; *p* = 0.006). Even after controlling for BMI, drinking behavior, cholesterol level, blood pressure, smoking, glucose levels, and comorbidity score, the inverse link persisted (aHR: 0.77, 95% CI: 0.66–0.90; *p* = 0.001).

**Figure 2:**
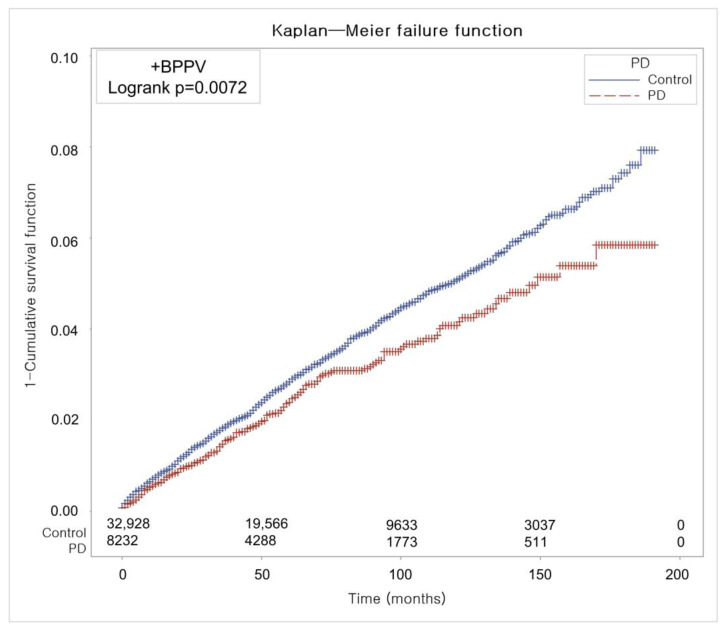
The cumulative risk of developing BPPV over 200 months, showing that patients with PD experienced significantly fewer cases than matched controls based on Kaplan–Meier analysis.

Figure 2 illustrates the time-to-event curve and cumulative incidence trajectory for BPPV during the 200-month observation period. According to the log-rank test, the cumulative incidence of BPPV was significantly lower in the PD group than in the control group (*p* = 0.0072).

### 3.3. Subgroup Analyses

Stratified subgroup analyses demonstrated that the tendency for PD to be linked with lower BPPV risk was consistently seen in various demographic subgroups. The association was particularly pronounced among individuals aged ≥75 years (aHR: 0.59, 95% CI: 0.43–0.82; *p* = 0.002). Both males (aHR: 0.73, 95% CI: 0.56–0.95; *p* = 0.019) and females (aHR: 0.80, 95% CI: 0.66–0.97; *p* = 0.024) with PD showed significantly lower risks of BPPV compared to their respective controls.

**Figure 3:**
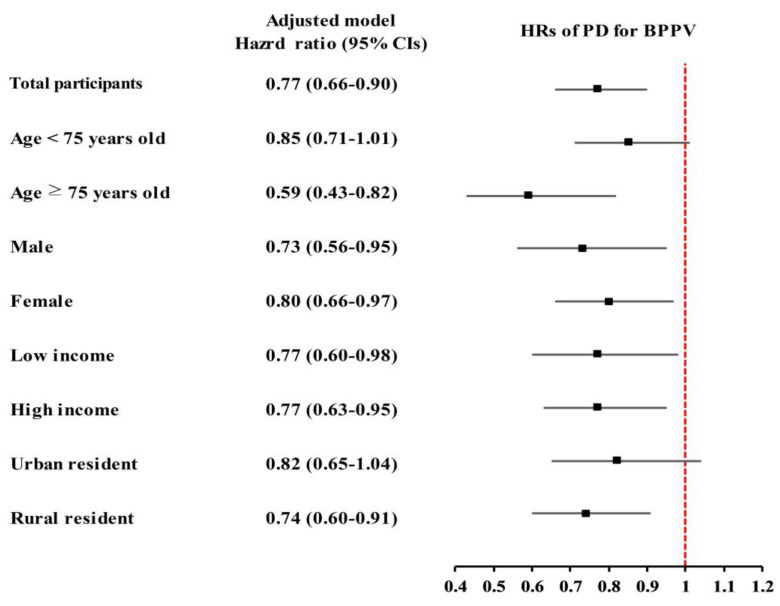
The inverse relationship between Parkinson’s disease (PD) and benign paroxysmal positional vertigo (BPPV) was consistently observed across various demographic subgroups, particularly among those aged ≥75 years. Both sexes, income levels, and rural residents demonstrated a lower risk of BPPV in the PD group, despite some subgroup results being similar but not statistically confirmed.

Similarly, the inverse association was observed in both low-income (aHR: 0.77, 95% CI: 0.60–0.98; *p* = 0.033) and high-income groups (aHR: 0.77, 95% CI: 0.63–0.95; *p* = 0.014), as well as among rural residents (aHR: 0.74, 95% CI: 0.60–0.91; *p* = 0.005). While not all subgroup findings reached statistical significance, similar trends were consistently observed across the remaining groups (Figure 3).

## 4. Discussion

In this nationwide longitudinal study, individuals with PD were found to be 23% less likely to develop BPPV than their matched counterparts. The observed relationship continued to show statistical significance following adjustment for cardiovascular confounders, metabolic parameters, and comorbidities. This study is among the first extensive, population-based cohort analyses to explore the longitudinal association between PD and incident BPPV, incorporating matched controls and thorough adjustment for confounding factors.

Previous studies have primarily examined dizziness and vestibular dysfunction as part of the broader PD symptom complex, without focusing specifically on BPPV as an outcome [16,24,32,35,36]. Early observational evidence from smaller studies suggests a potential co-occurrence of PD and BPPV; however, these lacked appropriate control groups, longitudinal follow-up, or were limited by small sample sizes [11,26]. Notably, one single-institution study (n = 305) found a slightly increased prevalence (5.3%) of BPPV among PD patients [26]. However, such findings may be constrained by sample bias and do not account for potential diagnostic overlap or under-reporting [26]. In contrast, our study may introduce a novel perspective by demonstrating a reduced risk of BPPV in PD patients, supported by robust methodology and nationwide representativeness.

Several biological, molecular, behavioral, and diagnostic mechanisms may help explain the inverse association observed between PD and BPPV. Neurodegeneration in PD—particularly involving the brainstem and vestibular pathways, such as the pedunculopontine nucleus and vestibular nuclei—may impair central vestibular processing [12,37]. This degeneration can blunt the responsiveness to positional changes that typically provoke BPPV symptoms [12,13,14], potentially contributing to their under-recognition or atypical manifestation in individuals with PD. Additionally, recent evidence suggests a shared oxidative stress pathway. Tsai et al. [24] reported elevated systemic oxidative stress in BPPV patients, with suppressed the activity of SIRT1—a neuroprotective histone deacetylase—leading to mitochondrial dysfunction and apoptosis. In vitro, SIRT1 inhibition in dopaminergic neuron-like cells exacerbated mitochondrial damage and neuronal apoptosis, mirroring PD-related neurodegeneration in BPPV models [24]. However, it is critical to interpret these findings with caution due to the retrospective nature of the dataset and the inherent diagnostic challenges in identifying BPPV in individuals with PD. Overlapping symptoms such as general dizziness, postural instability, and orthostatic hypotension may mask classical BPPV features. As a result, underdiagnosis is plausible and may partly explain the inverse association observed.

In addition to these central neurological changes, PD-related hypokinesia and reduced physical activity may also lower the risk of BPPV by limiting the mechanical triggers needed for otoconia displacement—a key factor in BPPV pathogenesis [36,38,39]. Otolith migration, which leads to canalithiasis or cupulolithiasis, is often precipitated by sudden or repetitive head movements [19,21]. However, due to bradykinesia, rigidity, and postural instability, PD patients typically engage in fewer of these movements [2,5], thereby reducing biomechanical stress on the vestibular system and lowering the likelihood of otoconia entering the semicircular canals [2,7,40]. Furthermore, dopaminergic modulation may influence vestibular function [41]. Dopaminergic projections from the substantia nigra to the vestibular nuclei may influence vestibular excitability and compensatory mechanisms [41]. As these pathways degenerate in PD, alterations in vestibular responses may occur. Some studies have also suggested that PD medications, such as levodopa, could impact balance and vestibular processing, although this area remains underexplored and warrants further investigation [42,43].

Beyond physiological mechanisms, diagnostic limitations might also contribute to the observed inverse association. In PD patients, reduced mobility, cognitive decline, and atypical symptom presentation can obscure classical features of BPPV—such as positional nystagmus observed during the Dix–Hallpike maneuver [11,12,13]. This diagnostic ambiguity may lead physicians to attribute dizziness symptoms to PD itself rather than investigating coexisting causes like BPPV, potentially resulting in under-reporting or missed diagnoses. This underdiagnosis is concerning given that BPPV is one of the most treatable causes of vertigo, with repositioning maneuvers offering rapid symptom relief. Failing to identify and treat BPPV may unnecessarily prolong discomfort and impair quality of life in PD patients. Supporting this notion, prior research has reported undetected BPPV in PD patients who had not sought care for dizziness [26], highlighting a potential diagnostic blind spot. Although subgroup analyses in our study revealed that the inverse association between PD and BPPV was consistent across sex and income levels—indicating that the relationship is not confined to specific demographic strata—the stronger association observed among individuals aged ≥75 and rural residents does not rule out the possibility of underlying disparities in healthcare access, clinical detection, or symptom reporting [36,44]. According to a 2019 OECD report, Korean rural regions—though geographically close to urban centers with a median travel time of only 25–30 min—continue to face challenges such as population aging, healthcare workforce shortages, and underutilization of diagnostic services [32]. These factors, combined with higher elderly dependency ratios in rural regions [32], might contribute to missed or delayed diagnoses of BPPV in older PD patients [12]. Taken together, neurodegenerative changes, reduced head movement, impaired mitochondrial resilience, and diagnostic limitations may collectively create a clinical context in which BPPV is either less likely to develop or less likely to be recognized in individuals with PD. Nevertheless, the consistent trends across all subgroups—along with statistical significance in most—support the interpretation of a genuine biological or behavioral association, further strengthened by the convergence of multidimensional mechanisms rather than being attributable to observational bias.

It is also important to consider that BPPV may be systematically under-recognized in PD due to atypical symptom presentation and the reduced likelihood of performing positional tests like the Dix–Hallpike maneuver in frail or cognitively impaired patients. Previous studies have identified undiagnosed BPPV in PD cohorts [26], and a Dutch study reported a 5.3% prevalence of BPPV among PD patients, suggesting potential under-reporting in routine clinical settings [26]. Therefore, the inverse association in our study may reflect limitations in diagnostic sensitivity rather than a true biological reduction in risk. Our findings may have important clinical implications. Although BPPV is less frequently diagnosed in patients with PD [26,36,38,39], it remains a highly treatable cause of vertigo, often responsive to simple canalith repositioning maneuvers [15,20]. The possibility of under-recognition due to atypical presentations underscores the need for increased clinician awareness. In patients with PD who report dizziness, maintaining a high index of suspicion for BPPV and incorporating routine vestibular assessments may facilitate earlier detection and treatment [26,45,46,47], ultimately improving quality of life [45,46,47].

This study has several limitations. First, diagnoses of PD and BPPV were based on administrative claims data using ICD-10 codes, which may be subject to misclassification or under-reporting, particularly in patients with mild or atypical presentations. Second, detailed clinical data such as dizziness duration, symptom severity, nystagmus findings, and treatment responses were not available. Third, although we adjusted for a broad set of covariates, residual confounding from unmeasured factors—such as physical activity levels, vestibular test results, or medication use—cannot be entirely ruled out. Fourth, the potential for underdiagnosis of BPPV in cognitively impaired or immobile PD patients warrants further prospective validation. Fifth, due to the limitations of the NHIS-HEALS dataset, we were unable to determine whether patients were drug-naïve at the time of PD diagnosis. To mitigate this, we applied a two-year wash-out period prior to the index date to reduce the risk of including preclinical PD cases. However, the lack of medication data remains a limitation in interpreting early clinical features. Additionally, the lack of clinical data on PD severity, such as Hoehn and Yahr stage or UPDRS scores [48], precluded analysis of the relationship between disease progression and BPPV incidence.

Despite these limitations, this study offers notable strengths. It utilizes a large, nationally representative cohort with long-term follow-up over 18 years. The comprehensive dataset includes demographic, socioeconomic, clinical, and lifestyle variables, allowing for detailed adjustment and subgroup analyses. The matched cohort design comprising 8232 individuals with PD and 32,928 matched controls, coupled with stratified Cox proportional hazards modeling, enhanced the validity of time-to-event comparisons and minimized selection bias [49,50]. Based on available literature, this is the first known epidemiological study to evaluate the PD–BPPV association at the national level with a rigorous methodological approach.

## 5. Conclusions

In this nationwide cohort study, patients with PD had a significantly lower risk of developing BPPV compared to matched controls. This inverse association remained consistent after adjusting for major confounders and was observed across various demographic subgroups which further supports the reliability and generalizability of the findings. Although the inverse association between PD and BPPV may reflect underlying behavioral and neurophysiological mechanisms, it is equally plausible that diagnostic barriers and symptom overlap have led to systematic underdiagnosis of BPPV in PD patients. Therefore, these results should not be interpreted as evidence of a causal or protective relationship, and further prospective studies with rigorous vestibular testing protocols are warranted, the results highlight the need for heightened clinical awareness of BPPV in PD patients, particularly given the condition’s high treatability and impact on patient quality of life. Incorporating vestibular assessments into routine evaluations of PD patients presenting with dizziness may improve detection and lead to better clinical outcomes. Future prospective studies with detailed clinical data are warranted to further elucidate the mechanisms underlying this association and to validate these observations.

## Figures and Tables

**Table 1:** Summary of baseline features of the participants.

Characteristics		Total Participants		
		Parkinson’s Disease	Control	Standardized Mean Difference
Age (years old) (n, %)				0.00
	40–44	5 (0.06)	20 (0.06)	
	45–49	64 (0.78)	256 (0.78)	
	50–54	220 (2.67)	880 (2.67)	
	55–59	481 (5.84)	1924 (5.84)	
	60–64	840 (10.20)	3360 (10.20)	
	65–69	1286 (15.62)	5144 (15.62)	
	70–74	1820 (22.11)	7280 (22.11)	
	75–79	1957 (23.77)	7828 (23.77)	
	80–84	1182 (14.36)	4728 (14.36)	
	85+	377 (4.58)	1508 (4.58)	
Sex (n, %)				0.00
	Male	3967 (48.19)	15,868 (48.19)	
	Female	4265 (51.81)	17,060 (51.81)	
Income (n, %)				0.00
	1 (lowest)	1527 (18.55)	6108 (18.55)	
	2	897 (10.90)	3588 (10.90)	
	3	1113 (13.52)	4452 (13.52)	
	4	1586 (19.27)	6344 (19.27)	
	5 (highest)	3109 (37.77)	12,436 (37.77)	
Residential region (n, %)				0.00
	Urban	3106 (37.73)	12,424 (37.73)	
	Rural	5126 (62.27)	20,504 (62.27)	
Weight status † (n, %)				0.06
	Underweight	300 (3.64)	1234 (3.75)	
	Normal	2911 (35.36)	11,868 (36.04)	
	Overweight	2157 (26.20)	8582 (26.06)	
	Obese I	2588 (31.44)	10,140 (30.79)	
	Obese II	276 (3.35)	1104 (3.35)	
Smoking status (n, %)				0.05
	Nonsmoker	6293 (76.45)	24,376 (74.03)	
	Past smoker	629 (7.64)	2618 (7.95)	
	Current smoker	1310 (15.91)	5934 (18.02)	
Alcohol consumption (n, %)				0.05
	<1 time a week	6527 (79.29)	25,434 (77.24)	
	≥1 time a week	1705 (20.71)	7494 (22.76)	
Systolic blood pressure (mmHg; mean, SD)		129.08 (17.35)	130.09 (17.00)	0.02
Diastolic blood pressure (mmHg; mean, SD)		78.33 (10.73)	78.33 (10.61)	0.00
Fasting blood glucose (mg/dL; mean, SD)		106.97 (36.54)	103.43 (29.47)	0.11
Total cholesterol (mg/dL; mean, SD)		193.73 (40.44)	195.91 (40.21)	0.05
CCI score ‡ (mean, SD)		1.30 (1.81)	1.00 (1.71)	0.17
BPPV (n, %)		193 (2.34)	1080 (3.28)	0.06

Abbreviations: SD, standard deviation; CCI, Charlson comorbidity index; BPPV, benign paroxysmal positional vertigo. † Weight status (BMI, body mass index, kg/m^2^) was categorized as <18.5 (underweight), ≥18.5 to <23 (normal), ≥23 to <25 (overweight), ≥25 to <30 (obese I), and ≥30 (obese II). ‡ The CCI score was calculated excluding diabetes (E10–E14) and cerebral stroke (I60–I66).

**Table 2:** Incidence rates and hazard ratios for BPPV in patients with PD and matched controls across subgroups.

		N of Event/ N of Total (%)	Follow-Up Duration (PY)	IR per 1000 (PY)	IRD (95% CI)	Hazard Ratios for BPPV			
						Crude †	*p*	Adjusted †‡	*p*
Total participants (n = 41,160)									
	PD	193/8232 (2.34)	38,784	4.98	−0.97 (−1.81–−0.14)	0.81 (0.69–0.94)	0.006 *	0.77 (0.66–0.90)	0.001 *
	Control	1080/32,928 (3.28)	181,367	5.95		1		1	
Age < 75 years old (n = 23,580)									
	PD	149/4716 (3.16)	26,615	5.60	−0.57 (−1.61–0.46)	0.88 (0.74–1.05)	0.158	0.85 (0.71–1.01)	0.070
	Control	759/18,864 (4.02)	122,982	6.17		1		1	
Age ≥ 75 years old (n = 17,580)									
	PD	44/3516 (1.25)	12,169	3.62	−1.88 (−3.29–−0.48)	0.63 (0.46–0.86)	0.004 *	0.59 (0.43–0.82)	0.002 *
	Control	321/14,064 (2.28)	58,385	5.50		1		1	
Male (n = 19,835)									
	PD	66/3967 (1.66)	16,931	3.90	−0.89 (−2.02–0.23)	0.80 (0.61–1.04)	0.090	0.73 (0.56–0.95)	0.019 *
	Control	398/15,868 (2.51)	83,012	4.79		1		1	
Female (n = 21,325)									
	PD	127/4265 (2.98)	21,853	5.81	−1.12 (−2.32–0.08)	0.81 (0.67–0.98)	0.030 *	0.80 (0.66–0.97)	0.024 *
	Control	682/17,060 (4.00)	98,355	6.93					
Low income group (n = 17,685)									
	PD	78/3537 (2.21)	16,570	4.71	−0.98 (−2.23–0.26)	0.80 (0.63–1.02)	0.070	0.77 (0.60–0.98)	0.033 *
	Control	450/14,148 (3.18)	79,042	5.69		1		1	
High income group (n = 23,475)									
	PD	115/4695 (2.45)	22,214	5.18	−0.98 (−2.10–0.14)	0.81 (0.66–0.99)	0.038 *	0.77 (0.63–0.95)	0.014 *
	Control	630/18,780 (3.35)	102,325	6.16		1		1	
Urban resident (n = 15,530)									
	PD	87/3106 (2.80)	14,852	5.86	−0.93 (−2.37–0.51)	0.84 (0.67–1.05)	0.127	0.82 (0.65–1.04)	0.100
	Control	470/12,424 (3.78)	69,246	6.79		1		1	
Rural resident (n = 25,630)									
	PD	106/5126 (2.07)	23,932	4.43	−1.01 (−2.02–0.00)	0.78 (0.64–0.96)	0.020 *	0.74 (0.60–0.91)	0.005 *
	Control	610/20,504 (2.98)	112,121	5.44		1		1	

Abbreviations: PD, Parkinson’s disease; BPPV, benign paroxysmal positional vertigo; IR, incidence rate; IRD, incidence rate difference; PY, person-years. * Stratified Cox proportional hazard regression model, significance at *p* < 0.05. † Crude model was stratified by age, sex, income, and region of residence. ‡ The adjusted model was adjusted for weight status, smoking, alcohol consumption, systolic blood pressure, diastolic blood pressure, fasting blood glucose, total cholesterol, and CCI scores.

## Data Availability

All data are available from the database of National Health Insurance Sharing Service (NHISS) https://nhiss.nhis.or.kr/ (accessed on 1 October 2024). NHISS allows access to all of these data for any researcher who promises to follow the research ethics at some processing charge. If you want to access the data of this article, you can download them from the website after promising to follow the research ethics.

## Abbreviations

The following abbreviations are used in this manuscript:

PD	Parkinson’s diseases
BPPV	benign paroxysmal positional vertigo
IRD	incidence rate difference
PY	person-years
CCI	Charlson comorbidity index
HR	hazard ratio
CI	confidence interval
